# Long Non-Coding RNA MAPK8IP1P2 Inhibits Lymphatic Metastasis of Thyroid Cancer by Activating Hippo Signaling *via* Sponging miR-146b-3p

**DOI:** 10.3389/fonc.2020.600927

**Published:** 2021-01-07

**Authors:** Xiaoli Liu, Qingfeng Fu, Xuehai Bian, Yantao Fu, Jingwei Xin, Nan Liang, Shijie Li, Yishen Zhao, Li Fang, Changlin Li, Jiao Zhang, Gianlorenzo Dionigi, Hui Sun

**Affiliations:** ^1^ Division of Thyroid Surgery, China–Japan Union Hospital of Jilin University, Jilin Provincial Key Laboratory of Surgical Translational Medicine, Changchun, China; ^2^ Division for Endocrine and Minimally Invasive Surgery, Department of Human Pathology in Adulthood and Childhood “G. Barresi”, University Hospital “G. Martino”, University of Messina, Messina, Italy

**Keywords:** thyroid cancer, lymph node metastasis, anoikis resistance, MAPK8IP1P2, Hippo signaling

## Abstract

The principal issue derived from thyroid cancer is its high propensity to metastasize to the lymph node. Aberrant exprssion of long non-coding RNAs have been extensively reported to be significantly correlated with lymphatic metastasis of thyroid cancer. However, the clinical significance and functional role of lncRNA-MAPK8IP1P2 in lymphatic metastasis of thyroid cancer remain unclear. Here, we reported that MAPK8IP1P2 was downregulated in thyroid cancer tissues with lymphatic metastasis. Upregulating MAPK8IP1P2 inhibited, while silencing MAPK8IP1P2 enhanced anoikis resistance *in vitro* and lymphatic metastasis of thyroid cancer cells *in vivo*. Mechanistically, MAPK8IP1P2 activated Hippo signaling by sponging miR-146b-3p to disrupt the inhibitory effect of miR-146b-3p on NF2, RASSF1, and RASSF5 expression, which further inhibited anoikis resistance and lymphatic metastasis in thyroid cancer. Importantly, miR-146b-3p mimics reversed the inhibitory effect of MAPK8IP1P2 overexpression on anoikis resistance of thyroid cancer cells. In conclusion, our findings suggest that MAPK8IP1P2 may serve as a potential biomarker to predict lymphatic metastasis in thyroid cancer, or a potential therapeutic target in lymphatic metastatic thyroid cancer.

## Introduction

Thyroid cancer is one of the most prevalent endocrine malignancies with an increasing incidence in recent years worldwide (1, 2). According to histological classification, thyroid carcinoma can be divided into papillary thyroid cancer (PTC), follicular thyroid cancer (FTC), anaplastic thyroid cancer (ATC), and medullary thyroid cancer (MTC), where PTC is the most common histological type, accounting for 85–90% of all cases (3). Most PTCs are effectively treated by surgical removal, followed by adjuvant radioactive iodine therapy, and has a favorable 5-year survival rate exceeding 95% (4). However, the principal issue derived from PTC is its high propensity to metastasize to lymph node, which significantly affects the prognosis of thyroid cancer patients (5). Therefore, identification of lymph node metastasis-relevant factor will facilitate early detection of lymph node metastasis and development of anti-lymph node metastasis therapeutic strategy in thyroid cancer patients.

With recent technological advances enabling us to detect rare circulating tumor cells that are anoikis resistant, anoikis resistance becomes a hot topic in cancer research. Anoikis resistance that is a kind of capacity of cancer cells to survive under suspension conditions has been extensively reported to be a hallmark of metastatic cancer cells, which significantly contributes to distant metastasis in various cancer types, including bone metastasis of prostate cancer (6) and multiple organs metastasis in non-small cell lung cancer (7). Furthermore, anoikis resistance has also been demonstrated to play an important role in metastatic thyroid cancer. Kittirat Saharat and colleagues have reported that tumor susceptibility gene 101 protein (TSG101) was identified to be upregulated in anoikis resistant thyroid cancer cells, which was accompanied with decreased expression of an apoptotic marker (cleaved poly-ADP ribose polymerase) and a pro-apoptotic protein (BCL-2 like protein 4) (8). Our previous study revealed that anoikis resistance induced by miR-424-5p promoted lung metastasis of thyroid cancer by inactivating Hippo signaling *via* simultaneously targeting WWC1, SAV1, and LAST2 (9). Recently, the critical role of anoikis resistance in lymphatic metastasis of cancer seizes great attention (10, 11). In several cancer scenario, anoikis resistance correlated significantly with positive lymph node metastasis status, including esophageal carcinoma (12, 13), melanoma (14), tongue cancer (15), breast cancer (16), and colorectal cancer (17). Importantly, development of anoikis resistance has been reported to significantly contribute to lymphatic metastasis of thyroid cancer (18–20). Therefore, investigating the underlying mechanism of anoikis resistance in lymphatic metastasis of thyroid cancer is of great necessity.

The long non-coding RNAs (lncRNAs) are a kind of newly discovered class of non-coding RNA with the length longer than 200 nucleotides (21, 22). They implicate several biological processes through various mechanisms, including transcriptional regulation as enhancers to modulate transcription of their target genes, post-transcription as decoys to bind proteins or scaffolds to regulate interactions between proteins and genes, and epigenetic modification as competing endogenous RNAs (ceRNA) to sponge target miRNAs so as to disrupt the miRNAs-mediated degradation of target genes (21, 22). A great deal of attention has focused on the role of lncRNAs in miRNA-mediated lncRNA/mRNA crosstalk (23), and dysregulation of miRNAs is inherently linked to the progression and metastasis of various types of cancer (9, 24–26). Recently, there is a great body of evidence reporting the role of lncRNAs in the development, progression, and metastasis in a number of cancers (27–29), including lymph node metastasis (30, 31). Notably, numerous studies have shown that aberrant expression of lncRNAs is significantly correlated with lymph node metastasis in thyroid cancer patients (32–34). Although these findings indicated that lncRNAs may hold clinical applicable value as the potential predictive markers for early detection of lymph node metastasis in thyroid cancer, whether lncRNAs affects lymph node metastasis of thyroid cancer *in vivo* is not determined in these studies. Therefore, further investigation of the functional role of lncRNA in lymph node metastasis of thyroid cancer *in vivo* will provide experimental evidence to support the applicable potential of lncRNAs to predict lymph node metastasis in thyroid cancer patients.

In the current study, we found that MAPK8IP1P2 was downregulated in thyroid cancer tissues, and particularly in thyroid cancer tissues with lymphatic metastasis, which was correlated with poor progression-free survival in thyroid cancer patients. Gain and loss of function assays showed that upregulating MAPK8IP1P2 inhibited, while silencing MAPK8IP1P2 enhanced anoikis resistance *in vitro* and lymphatic metastasis of thyroid cancer cells *in vivo*. Mechanistic investigations revealed that MAPK8IP1P2 activated Hippo signaling by sponging miR-146b-3p to disrupt the inhibitory effect of miR-146b-3p on NF2, RASSF1, and RASSF5 expression, which further inhibited anoikis resistance and lymphatic metastasis in thyroid cancer. Taken together, our findings provide the experimental evidence regarding the clinical significance and biological role of MAPK8IP1P2 in lymphatic metastasis of thyroid cancer, suggesting that MAPK8IP1P2 may be used as a potential biomarker to predict lymphatic metastasis in thyroid cancer patients.

## Materials and Methods

### Cell Lines and Cell Culture

Normal primary thyroid follicular epithelial cells (PTFE) were purchased from Procell (Procell Life Science & Technology Co., Ltd., Wuhan, China). Thyroid cancer cell lines, including PTC cell lines (B-CPAP and KTC-1) and anaplastic thyroid cancer (ATC) cell lines (BHT-101, CAL-62, KMH-2, and 8305C), were obtained from Cell Bank of Shanghai Institute of Cell Biology, Chinese Academy of Sciences (Shanghai, China). PTFE were cultured in CM-H023 medium (Procell, China), and thyroid cancer cell lines were cultured in RPMI-1640 medium (Life Technologies, Carlsbad, CA, USA) supplemented with penicillin G (100 U/ml), streptomycin (100 mg/ml), and 10% fetal bovine serum (FBS, Life Technologies). All cell lines were cultured at 37°C in a humidified atmosphere with 5% CO_2_.

### Patients and Tumor Tissues

The total of 48 fresh thyroid cancer tissues and 24 adjacent normal tissues were obtained during surgery at the China-Japan Union Hospital of Jilin University (Changchun, China) between January 2018 and December 2018 (**Table 1**). Patients were diagnosed based on clinical and pathological evidence, and the specimens were immediately snap-frozen and stored in liquid nitrogen tanks. For the use of these clinical materials for research purposes, prior patients’ consents and approval from the Institutional Research Ethics Committee were obtained (approval number #: 2019-NSFC-026).

**Table 1:** The basic information of 48 thyroid carcinoma patients for MAPK8IP1P2 RNA expression analysis.

		Cases (n)	Percentage (%)
Histologic	PTC	48	100.0
	Other	0	0.0
Gender	Male	1	2.1
	Female	47	97.9
Age	<50	41	85.4
	≥50	7	14.6
T classification	T1	22	45.8
	T2	15	31.2
	T3	9	18.8
	T4	2	4.2
N classification	N0	10	20.8
	N1	38	79.2
M classification	M0	48	100.0
	M1	0	0.0

*PTC, papillary thyroid carcinoma.

### Plasmid and Transfection

Human MAPK8IP1P2 cDNA (Vigene Biosciences, Shandong, China) was cloned into the pcDNA3.1(+) plasmid. Knockdown of endogenous MAPK8IP1P2 was performed by cloning two short hairpin RNA (shRNA) oligonucleotides into the GV493 vector (GenChem, Shanghai, China). The sequences of the two separate shRNA fragments are listed in **Table 2**. The 3′UTR regions of NF2, RASSF1, RASSF5, and the region including MAPK8IP1P2 sequence targeted by miR-146b-3p were PCR-amplified from genomic DNA and cloned into pmirGLO vectors (Promega, USA), and the list of primers used in cloning reactions was provided in **Table 2**. miR-146b-3p mimics were synthesized and purified by RiboBio. Transfection of plasmids was performed as previously described (35).

**Table 2:** A list of primers used in the reactions for clone PCR.

Gene	Sequence (5` – 3`)
shMAPK8IP1P2-1#-up	CCGGGCAGTTTCACAAGCAGTTTCTCGAGAAACTGCTTGTGAAACTGCTTTTTTG
shMAPK8IP1P2-1#-dn	AATTCAAAAAGCAGTTTCACAAGCAGTTTCTCGAGAAACTGCTTGTGAAACTGCT
shMAPK8IP1P2-2#-up	CCGGCCGGACCATATTCAGGTTTCTCGAGAAACCTGAATATGGTCCGGTTTTTTG
shMAPK8IP1P2-2#-dn	AATTCAAAAACCGGACCATATTCAGGTTTCTCGAGAAACCTGAATATGGTCCGGT
MAPK8IP1P2-up	ATAGGTGTCAAGGCCGATGACTC
MAPK8IP1P2-dn	CGCAGATGACAGAGCTGAGAAC
NF2-3`UTR-up	CTCTCATGGCGTTCTAGTTCTCTG
NF2-3`UTR-dn	AAAGTGAGGCCTGGGTACAAC
RASSF1-3`UTR-up	TTGTACCCCCAGGTGGAAGG
RASSF1-3`UTR-dn	GATGATGACTGTCACCCCAACC
RASSF5-3`UTR-up	CCTGGAAAAAGAGGAGCAGGAC
RASSF5-3`UTR-dn	TCTGAGCCAGCCTCAGCTTTG

### RNA Extraction, Reverse Transcription, and Real-Time PCR

RNA from tissues and cells was extracted (TRIzol, Life Technologies) according to the manufacturer’s instructions. Messenger RNA (mRNA), lncRNA, and miRNA were reverse transcribed from the total RNA using the Revert Aid First Strand cDNA Synthesis Kit (Thermo, USA) according to the manufacturer’s protocol. Complementary DNA (cDNA) was amplified and quantified on ABI 7500HT system (Applied Biosystems, Foster City, CA, USA) using SYBR Green I (Applied Biosystems). The primers used in the reactions are listed in **Table 3**. Primers for U6 and miR-146b-3p were synthesized and purified by RiboBio (Guangzhou, China). Real-time PCR was performed as described previously (36). Glyceraldehyde-3-phosphate dehydrogenase (GAPDH) was used as the endogenous controls for mRNA and lncRNA, and U6 was used as the endogenous control for miRNA. Relative fold expressions were calculated with the comparative threshold cycle (37).

**Table 3:** A list of primers used in the reactions for real-time RT-PCR.

Gene	Sequence (5`– 3`)
MAPK8IP1P2-up	GGGAGAGCATTCCAGCAGTTTC
MAPK8IP1P2-dn	TCCTCAAGCAGTGCCACATC
GAPDH-up	TCCTCTGACTTCAACAGCGACAC
GAPDH-dn	CACCCTGTTGCTGTAGCCAAATTC
CTGF-up	TGGAGATTTTGGGAGTACGG
CTGF-dn	CAGGCTAGAGAAGCAGAGCC
CYR61-up	GGTCAAAGTTACCGGGCAGT
CYR61-dn	GGAGGCATCGAATCCCAGC
HOXA1-up	TCCTGGAATACCCCATACTTAGC
HOXA1-dn	GCACGACTGGAAAGTTGTAATCC
SOX9-up	AGCGAACGCACATCAAGAC
SOX9-dn	CTGTAGGCGATCTGTTGGGG
RPL13A-up	GCCATCGTGGCTAAACAGGTA
RPL13A-dn	GTTGGTGTTCATCCGCTTGC
PPIA-up	GGCAAATGCTGGACCCAACACA
PPIA-dn	TGCTGGTCTTGCCATTCCTGGA
NF2-up	AGTGGCCTGGCTCAAAATGG
NF2-dn	TGTTGTGTGATCTCCTGAACCA
RASSF1-up	AGGACGGTTCTTACACAGGCT
RASSF1-dn	TGGGCAGGTAAAAGGAAGTGC
RASSF5-up	GGGCATGAAACTGAGTGAAGA
RASSF5-dn	TGGCATCATAGATGGACTGGG

### Western Blotting Analysis

Western blot was performed according to a standard method, as described previously (38). Antibodies against BAX (#5023), BAD (#9239), BCL2L1 (#2764), BCL2 (#2872), p-MST1 (Thr183)/2(Thr180) (#3681), MST1 (#14946), p-LATS1(Thr1079) (#8654), LATS1 (#9153), p-YAP(Ser127) (#13008), YAP (#14074), NF2 (#6995), and RASSF1 (#86026) were purchased from Cell Signaling Technology, and TAZ from Abcam (ab224239), RASSF5 from Sigma (N5912). The membranes were stripped and reprobed with an anti–α-tubulin antibody (Cell Signaling Technology) as the loading control.

### Anchorage-Independent Growth Assay

Five hundred cells were trypsinized and resuspended in complete medium containing 0.3% agar (Sigma). This experiment was performed as previously described (39) and carried out three times independently for each cell line.

### Cell Counting Kit-8 Analysis

Next, 2 × 10^3^ cells were seeded into 96 well plates and the specific staining process and methods were performed according to the previous study (40).

### Colony Formation Assay

The cells were trypsinized as single cell and suspended in the media with 10% FBS. The indicated cells (300 cells per well) were seeded into of 6-well plate for ~10–14 days. Colonies were stained with 1% crystal violet for 10 min after fixation with 10% formaldehyde for 5 min. Plating efficiency was calculated as previously described (41). Different colony morphologies were captured under a light microscope (Olympus).

### Cell Cycle Analysis

Pretreatment and staining was performed using Cell Cycle Detection Kit (KeyGEN, China) as previously described (42). Briefly, cells (5 × 10^5^) were harvested by trypsinization, washed in ice-cold phosphate-buffered saline (PBS), and fixed in 75% ice-cold ethanol in PBS. Before staining, cells were gently resuspended in cold PBS, and ribonuclease was added into cells’ suspension tube incubated at 37°C for 30 min, followed by incubation with propidium iodide (PI) for 20 min at room temperature. Cell samples (2 × 10^4^) were then analyzed by FACSCanto II flow cytometer (Becton, Dickinson and Company, Franklin Lakes, NJ, USA) and the data were analyzed using FlowJo 7.6 software (TreeStar Inc., Ashland, OR, USA).

### Anoikis Induction Assay

Cell culture plates were coated with poly-HEMA (P3932; Sigma-Aldrich, St. Louis, USA), a non-adhesive substratum, and allowed to evaporate to dryness at room temperature. Cells were kept in suspension by using poly-HEMA coated plates to prevent adhesion. After 48 h of suspension, cells were harvested for cell viability analysis by 3-(4,5-dimethyl-2-thiazolyl)-2,5-diphenyl-2-H-tetrazolium bromide (MTT) assay and cell apoptosis analysis by flow cytometry.

### Annexin V Apoptosis Detection

Flow cytometric analyzed of apoptosis were using the FITC Annexin V Apoptosis Detection Kit I (BD, USA), and performed as previously described (43). The cell’s inner mitochondrial membrane potential (Δψm) was detected by flow cytometric using MitoScreen JC-1 staining kit (BD) (44). Briefly, cells were dissociated with trypsin and resuspended at 1 × 10^6^ cells/ml in Assay Buffer, and then incubated at 37°C for 15 minutes with 10 μl/ml JC-1. Before analyzed by flow cytometer, cells were washed twice by Assay Buffer. Flow cytometry data were analyzed using FlowJo 7.6 software (TreeStar Inc., USA) as previously described (45).

### Caspase-9 or Caspase-3 Activity Assays

Activity of caspase-9 or caspase-3 was analysis by spectrophotometry using Caspase-9 Colorimetric Assay Kit or Caspase-3 Colorimetric Assay Kit (Keygen, China), and was presented as protocol described. Briefly, 5 × 10^6^ cells or 100 mg fresh tumor tissues were washed with cold PBS and resuspended in Lysis Buffer and incubated on ice for 30 min, then mixed the 50 μl cell suspension, 50 μl Reaction Buffer, and 5 μl Caspase-3/-9 substrate, and then incubated at 37°C for 4 hours. The absorbance was measured at 405 nm, and BCA protein quantitative analysis was used as the reference to normal each experiment groups.

### Animal Study

Eight-week-old BALB/c-nu mice were purchased from the Experimental Animal Center of the Guangzhou University of Chinese Medicine and housed as previously described (46). The mice were randomly divided into three groups (n = 6 per group) and the indicated K1 cells (1 × 10^6^) were injected into footpad of mice. The primary tumors were allowed to form, then the mice were euthanized on the end-points, and the inguinal lymph nodes were excised and paraffin embedded. Sections of the lymph nodes were subjected to H & E staining for histological examination, and the tumor cell number was counted as previously described (7). Animal study was approved from the Institutional Research Ethics Committee of Jilin University, and approval number was KT201902051.

### Luciferase Assay

Cells (4 × 10^4^) were seeded in triplicate in 24-well plates and cultured for 24 h, and the luciferase reporter assay was performed as previously described (47). Cells were transfected with 100 ng HOP-Flash (Catalog # 83467, Addgene) or HIP-Flash luciferase reporter plasmid (Catalog # 83466, Addgene), plus 5 ng pRL-TK Renilla plasmid (Promega) using Lipofectamine 3000 (Invitrogen) according to the manufacturer’s recommendation. Luciferase and Renilla signals were measured 36 h after transfection using a Dual Luciferase Reporter Assay Kit (Promega) according to the manufacturer’s protocol.

### Statistical Analysis

All values are presented as means ± standard deviation (SD). Significant differences were determined using GraphPad 5.0 software (USA). Student’s t-test was used to determine statistical differences between two groups. One-way ANOVA was used to determine statistical differences between multiple testing. Survival curves were plotted using the Kaplan Meier method and compared by log-rank test. P < 0.05 was considered significant. All the experiments were repeated three times.

## Results

### MAPK8IP1P2 Is Downregulated in Thyroid Cancer With Lymph Node Metastasis

**Figure 1:**
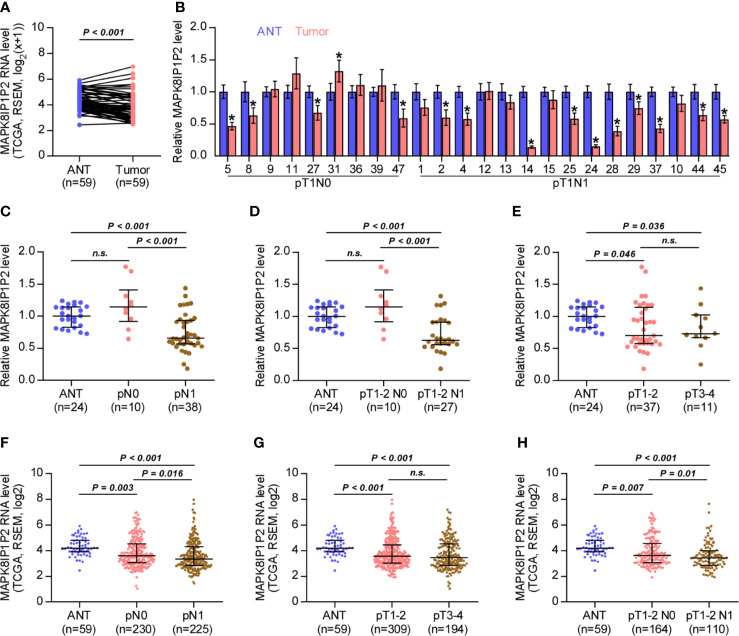
MAPK8IP1P2 is downregulated in thyroid cancer with lymphatic metastasis. **(A)** MAPK8IP1P2 expression in 59 paired thyroid cancer tissues and the matched adjacent normal tissues in the thyroid cancer dataset from TCGA. **(B)** Real-time PCR analysis of MAPK8IP1P2 expression in our 24 paired thyroid cancer tissues and their matched adjacent normal tissues, including 9 thyroid cancer tissues without lymphatic metastasis and 15 thyroid cancer tissues with lymphatic metastasis. The number on the abscissa indicated the patient number according to our record when collecting patient information. GAPDH was used as endogenous controls. *P < 0.05. **(C)** Real-time PCR analysis of MAPK8IP1P2 expression in ANT (n = 24), thyroid cancer tissues without lymphatic metastasis (n = 10), and thyroid cancer tissues with lymphatic metastasis (n = 38). GAPDH was used as endogenous controls. n.s. indicates no significance. **(D)** Real-time PCR analysis of MAPK8IP1P2 expression in ANT (n = 24), thyroid cancer tissues of T1-T2 grade without lymphatic metastasis (n = 10), and thyroid cancer tissues of T1-T2 grade with lymphatic metastasis (n = 27). GAPDH was used as endogenous controls. n.s. indicates no significance. **(E)** Real-time PCR analysis of MAPK8IP1P2 expression in ANT (n = 24), thyroid cancer tissues with T1-T2 grade (n = 37), and thyroid cancer tissues with T3-T4 grade (n = 11). GAPDH was used as endogenous controls. n.s. indicates no significance. **(F)** MAPK8IP1P2 expression in ANT (n = 59), thyroid cancer tissues without lymphatic metastasis (n = 230), and thyroid cancer tissues with lymphatic metastasis (n = 225) in the thyroid cancer dataset from TCGA. **(G)** MAPK8IP1P2 expression in ANT (n = 59), thyroid cancer tissues with T1-T2 grade (n = 309), and thyroid cancer tissues with T3-T4 grade (n = 194) in the thyroid cancer dataset from TCGA. **(H)** MAPK8IP1P2 expression in ANT (n = 59), thyroid cancer tissues of T1-T2 grade without lymphatic metastasis (n = 164), and thyroid cancer tissues of T1-T2 grade with lymphatic metastasis (n = 110) in the thyroid cancer dataset from TCGA.

By analyzing RNA sequencing dataset of thyroid cancer from The Cancer Genome Atlas (TCGA), we found that MAPK8IP1P2 was marked downregulated in thyroid cancer tissues compared with that in the adjacent normal tissues (ANT) (**Figure 1A**). Consistently, MAPK8IP1P2 expression was reduced in our 24 paired thyroid cancer tissues compared with the matched ANT (**Figure 1B**). Interestingly, we found that downexpression of MAPK8IP1P2 occurred in 4/9 (44.4%) thyroid cancer tissues without lymph node metastasis, and was 10/15 (66.7%) in thyroid cancer tissues with lymph node metastasis (**Figure 1B**). Our results further indicated that MAPK8IP1P2 expression in thyroid cancer tissues without lymph node metastasis had no significant difference compared with that in ANT (**Figures 1C, D**), but was dramatically and significantly downregulated in thyroid cancer tissues with lymph node metastasis (**Figure 1C**), even in lymph node metastatic thyroid cancer tissues with T1-T2 (**Figure 1D**). However, there was no significant difference of MAPK8IP1P2 expression between in T1-2 thyroid cancer tissues and in T3-4 thyroid cancer tissues, although MAPK8IP1P2 was downregulated in both compared with that in ANT (**Figure 1E**). These findings suggested that downexpression of MAPK8IP1P2 may play an important role in lymphatic metastasis of thyroid cancer. TCGA analysis further supported this finding that MAPK8IP1P2 expression was reduced in thyroid cancer tissues compared with that in ANT, especially in thyroid cancer tissues with lymph node metastasis (**Figures 1F–H**). Therefore, our results combined with TCGA analysis suggest that downexpression of MAPK8IP1P2 may be implicated in lymphatic metastasis of thyroid cancer.

### Upregulating MAPK8IP1P2 Inhibits Lymphatic Metastasis *In Vivo*

**Figure 2:**
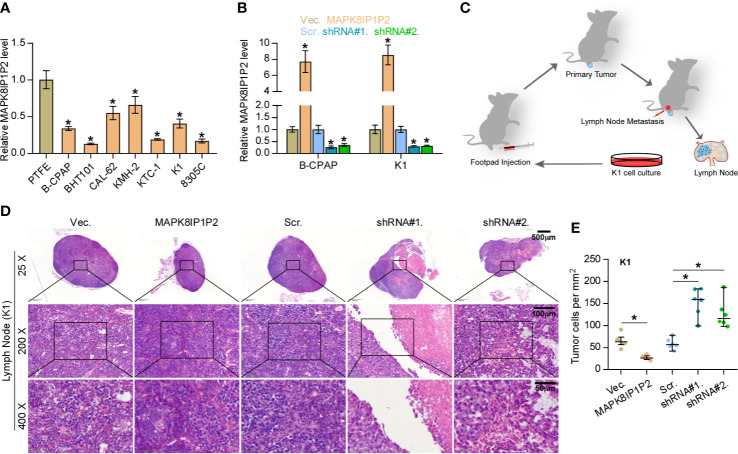
Upregulating MAPK8IP1P2 inhibits cancer stem cell characteristics in thyroid cancer cells. **(A)** Real-time PCR analysis of MAPK8IP1P2 expression in 7 thyroid cancer cells, including 4 PTC cell lines, B-CPAP, BHT101, KTC-1, and K1, and 2 ATC cell lines, CAL-62 and 8305C, and 1 thyroid duct cell carcinoma cells, TT, and a normal thyroid follicular epithelial cell line PTFE. GAPDH was used as endogenous controls. *P < 0.05. **(B)** MAPK8IP1P2 expression in the vector, MAPK8IP1P2 overexpression scramble, MAPK8IP1P2 shRNA#1, and MAPK8IP1P2 shRNA#2 thyroid cancer cells using real-time PCR. Transcript levels were normalized by GAPDH expression. *P < 0.05. **(C)** Schematic model of lymphatic metastasis model *in vivo*. **(D)** H & E staining analysis of tumors in lymph node from the indicated mice group. **(E)** The count of tumor cells in the tumor areas of lymph node from the indicated mice group. *P < 0.05.

Then, the effect of MAPK8IP1P2 on lymphatic metastasis of thyroid cancer cells *in vivo* was further investigated using the inguinal lymph node metastasis model. First, the expression levels of MAPK8IP1P2 in 7 thyroid cancer cell lines and a normal thyroid follicular epithelial cell line PTFE were measured. As shown in **Figure 2A**, MAPK8IP1P2 was differentially downregulated in thyroid cancer cells compared with that in PTFE cells. We further constructed MAPK8IP1P2-stably overexpressing B-CPAP and K1 cells and endogenously knocked down MAPK8IP1P2 expression in B-CPAP and K1 cells, both of which expressed moderate levels of MAPK8IP1P2 compared with that in other thyroid cancer cell lines (**Figure 2B**). Then, Vector-, MAPK8IP1P2-overexpressing, scramble and MAPK8IP1P2-downexpressing K1 cells were injected into the surrounding tissues in the foot pads of the mice (n = 6/group) using the inguinal lymph node metastasis model (**Figure 2C**). The metastatic inguinal lymph nodes were excised after 4 weeks and analyzed by H & E staining. As show in **Figures 2D, E**, histological examination of the lymph node metastatic tumors revealed that the tumors in lymph nodes formed from MAPK8IP1P2-overexpressing cells exhibited reduce tumor burden and the decreased number of tumor cells compared with those injected with the vector-control cells. Conversely, the tumors in lymph nodes formed from MAPK8IP1P2-downexpressing cells had larger tumor burden and more number of tumor cells than those inoculated with the scramble cells (**Figures 2D, E**). These findings indicate that upregulating MAPK8IP1P2 inhibits lymphatic metastasis *in vivo*.

### Upregulating MAPK8IP1P2 Improves Anoikis Resistance in Thyroid Cancer Cells

**Figure 3:**
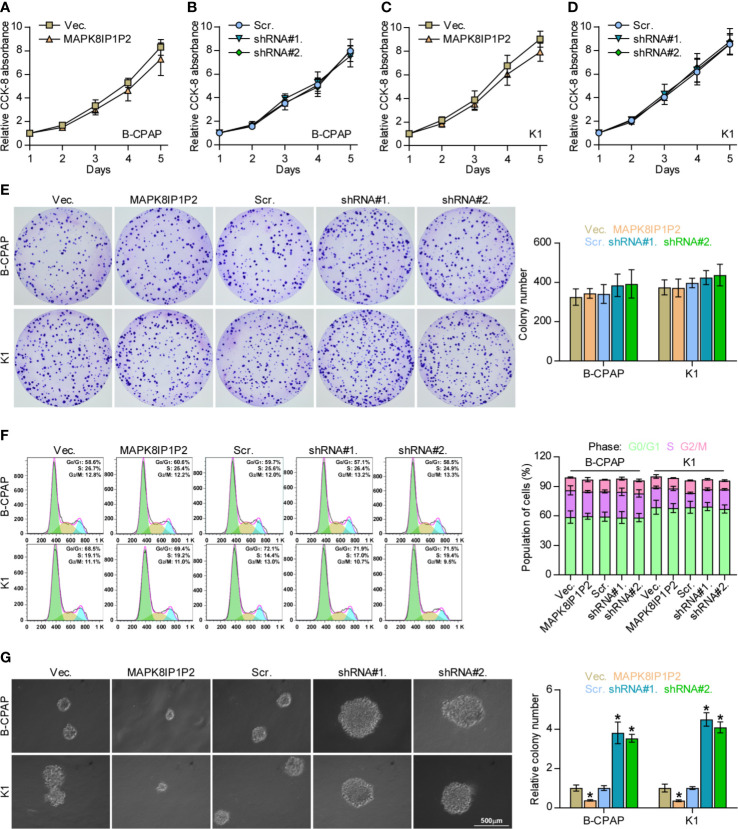
Upregulating MAPK8IP1P2 does not affect proliferation of thyroid cancer cells. **(A–D)** The effect of overexpression or silencing MAPK8IP1P2 on the cell growth in the indicated thyroid cancer cells by CCK-8 assay. **(E)** The effect of overexpression or silencing MAPK8IP1P2 on colony-formation ability of the indicated thyroid cancer cells by colony-formation assay. **(F)** The effect of overexpression or silencing MAPK8IP1P2 on cell cycle progression of the indicated thyroid cancer cells by flow cytometry. **(G)** The effect of overexpression or silencing MAPK8IP1P2 on survival ability in the indicated thyroid cancer cells by anchorage-independent growth assay. *P < 0.05.

The biological function of MAPK8IP1P2 in lymphatic metastasis of thyroid cancer was further CCK-8 assay showed that either upregulating or downregulating MAPK8IP1P2 had no significant effect on the cell growth of B-CPAP and K1 cells (**Figures 3A–D**). Similarly, neither colony-formation ability nor cell cycle progression was impeded by the changed expression of MAPK8IP1P2 in thyroid cancer cells (**Figures 3E, F**). However, upregulating MAPK8IP1P2 inhibited, while silencing MAPK8IP1P2 increased anchorage-independent growth capability of thyroid cancer cells (**Figure 3G**). These results indicate that the proliferation ability of thyroid cancer cells was not impeded by MAPK8IP1P2 *in vitro*.

**Figure 4:**
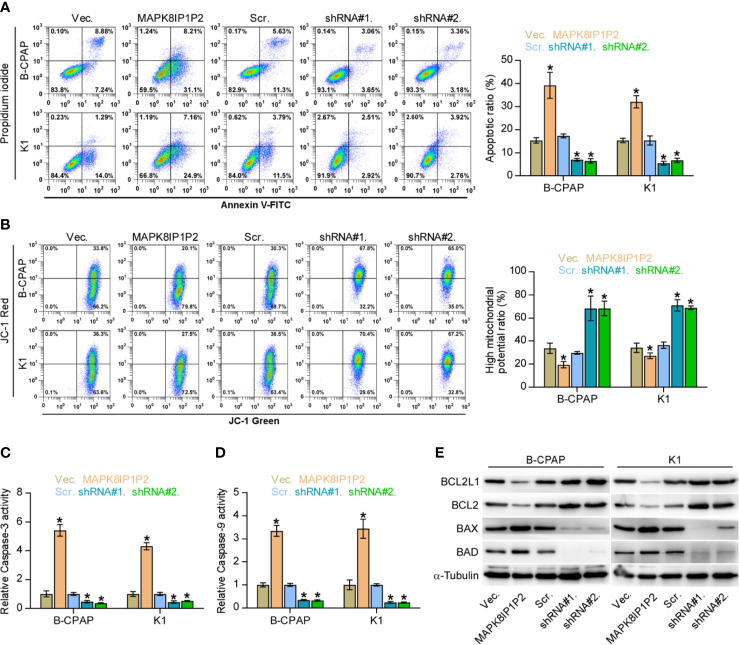
Upregulating MAPK8IP1P2 inhibits anoikis resistance in thyroid cancer cells. **(A)** The effect of overexpression or silencing MAPK8IP1P2 on the apoptotic ratio in the indicated thyroid cancer cells by Annexin V-FITC/PI staining. *P < 0.05. **(B)** The effect of overexpression or silencing MAPK8IP1P2 on mitochondrial potential in the indicated thyroid cancer cells by JC-1 staining. *P < 0.05. **(C, D)** The effect of overexpression or silencing MAPK8IP1P2 on the activities of caspase-3 **(C)** and caspase-9 **(D)** in the indicated thyroid cancer cells. *P < 0.05. **(E)** Western blotting analysis of the effect of overexpression or silencing MAPK8IP1P2 on anti-apoptotic proteins, BCL2 and BCL2L1, and pro-apoptotic proteins, BAD and BAX, in the indicated thyroid cancer cells. α-Tubulin served as the loading control.

Notably, upregulating MAPK8IP1P2 represses anchorage-independent growth capability of thyroid cancer cells as demonstrated above. Accumulating studies have shown that the capacity of cancer cells to survive under suspension conditions, namely anoikis resistance, is an important characteristic contributing to tumor progression and metastasis (6, 48, 49), including thyroid cancer (9, 28). Therefore, the effect of MAPK8IP1P2 on anoikis resistance in thyroid cancer cells was further evaluated. As shown in **Figure 4A**, upregulating MAPK8IP1P2 enhanced, while silencing MAPK8IP1P2 reduced the apoptosis rate of thyroid cancer cells. Mitochondrial potential assay showed that upregulating MAPK8IP1P2 attenuated, while silencing MAPK8IP1P2 elevated the mitochondrial potential of thyroid cancer cells (**Figure 4B**). The results of caspase activity assay and western blot analysis revealed that upregulating MAPK8IP1P2 increased the activity of caspase-3 or -9 and expression of pro-apoptotic proteins BAD and BAX, but reduced expression of anti-apoptotic proteins BCL2 and BCL2L1 (**Figures 4C–E**); conversely, silencing MAPK8IP1P2 yielded the opposite effect in thyroid cancer cells (**Figures 4C–E**). Taken together, our results indicate that upregulating MAPK8IP1P2 abrogates anoikis resistance in thyroid cancer cells.

### MAPK8IP1P2 Activates Hippo Signaling Pathway in Thyroid Cancer Cells

**Figure 5:**
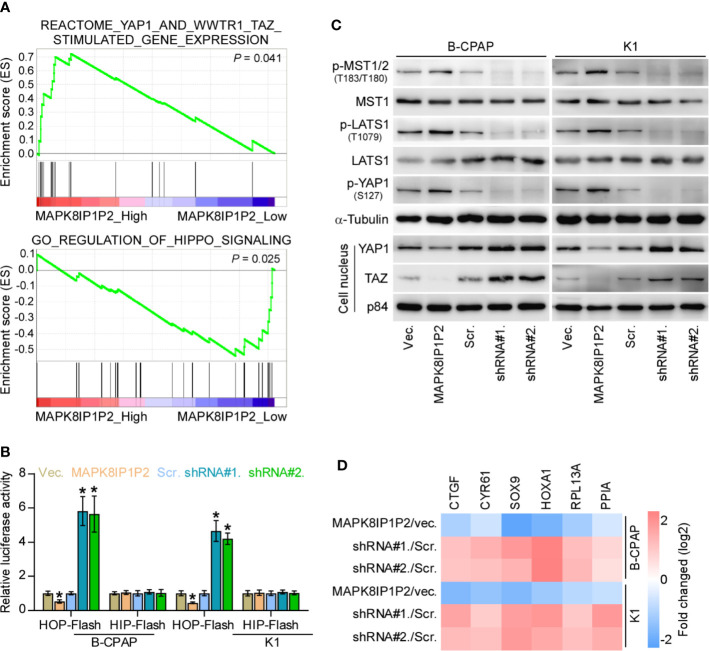
MAPK8IP1P2 activates Hippo signaling pathway in thyroid cancer cells. **(A)** Gene set enrichment analysis (GSEA) revealed that MAPK8IP1P2 expression positively correlated with Hippo signaling. **(B)** The effect of overexpression or silencing MAPK8IP1P2 on TEAD transcriptional activity was assessed by HOP-Flash luciferase reporter in the indicated cells. Error bars represent the mean ± S.D. of three independent experiments. *P < 0.05. **(C)** Western blotting analysis of the effect of overexpression or silencing MAPK8IP1P2 on phophorylated MST1/2 (p-MST1/2), phophorylated LATS1 (p-LATS1), phophorylated YAP1 (p-YAP1), total levels of MST1 and LATS1 and nuclear translocation of YAP1 and TAZ in the indicated thyroid cancer cells. α-Tubulin and p84 were served as the cytoplasmic and nuclear loading control respectively. **(D)** Real-time PCR analysis of the effect of overexpression or silencing MAPK8IP1P2 on CTGF, CYR61, HOXA1, PPIA, RPL13A, and SOX9 in the indicated cells. Transcript levels were normalized by GAPDH expression. Error bars represent the mean ± S.D. of three independent experiments. *P < 0.05.

To determine the underlying mechanism implicated in anti-lymphatic metastatic role of MAPK8IP1P2 in thyroid cancer, Gene Set Enrichment Analysis (GSEA) was performed based on MAPK8IP1P2 expression in the thyroid cancer dataset from TCGA. As shown in **Figure 5A**, MAPK8IP1P2 overexpression was positively correlated with activity of Hippo signaling pathway, but negatively associated with the transcriptional activity of downstream co-activators YAP1/TAZ of Hippo signaling. Inactivation of Hippo signaling has been widely reported to be implicated in anoikis resistance and metastatic thyroid cancer (9, 50, 51), as well as in lymphatic metastasis process of cancers (52, 53), suggesting that Hippo signaling may mediate the functional role of MAPK8IP1P2 in lymphatic metastasis of thyroid cancer. Luciferase reporter assay showed that upregulating MAPK8IP1P2 reduced, while silencing MAPK8IP1P2 increased the luciferase reporter activity of HOP-Flash, but not the HIP-Flash (**Figure 5B**), suggesting that upregulating MAPK8IP1P2 inhibits the TEAD-dependent luciferase activity in thyroid cancer cells. Furthermore, upregulating MAPK8IP1P2 enhanced the expression of phophorylated MST1/2 (p-MST1/2), phophorylated LATS1 (p-LATS1) and phophorylated YAP1 (p-YAP1), reduced the nuclear translocation of YAP1 and TAZ, but had no effect on total level of MST1 and LATS1 in thyroid cancer cells (**Figure 5C**). In contrast, silencing MAPK8IP1P2 reduced p-MST1/2, p-LATS1, and p-YAP1 expression, and increased the nuclear expression of YAP1 and TAZ (**Figure 5C**). Real-time PCR analysis showed that upregulating MAPK8IP1P2 decreased, while silencing MAPK8IP1P2 increased the expression levels of multiple downstream genes of Hippo pathway, including CTGF, CYR61, HOXA1, PPIA, RPL13A, and SOX9 (54, 55), in thyroid cancer cells (**Figure 5D**). Therefore, these findings indicate that MAPK8IP1P2 activates Hippo signaling in thyroid cancer cells.

### MAPK8IP1P2 Activates Hippo Signaling by Sponging miR-146b-3p

**Figure 6:**
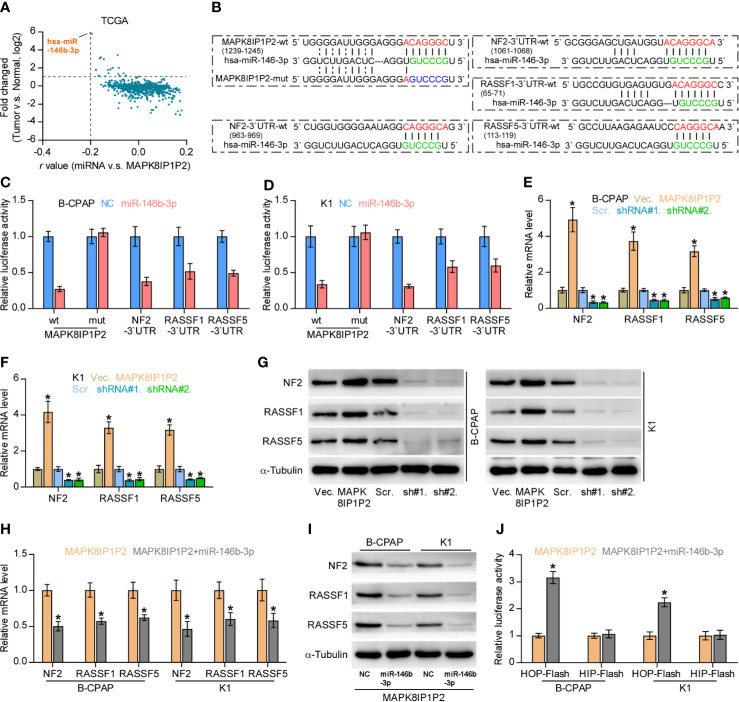
MAPK8IP1P2 activates Hippo signaling by sponging miR-146b-3p. **(A)** Volcano plot analyzed the clinical correlation of MAPK8IP1P2 with all reported miRNAs in thyroid cancer dataset from TCGA. The orange colors represent significantly and negatively correlated miRNAs with fold change > 2 and r value < -0.2. **(B)** Predicted recognition sites of miR-146b-3p on MAPK8IP1P2, and predicted miR-146b-3p targeting sequence and mutant sequences in 3’UTR s of NF2, RASSF1, and RASSF5. **(C, D)** The effect of miR-146b-3p on the luciferase activity of wild-type or mutant MAPK8IP1P2, NF2, RASSF1 and RASSF5 in the indicated cells. Error bars represent the mean ± S.D. of three independent experiments. *P < 0.05. **(E, F)** Real-time PCR analysis of the effect of overexpression or silencing MAPK8IP1P2 on NF2, RASSF1, and RASSF5 expression in the indicated cells. Transcript levels were normalized by GAPDH expression. Error bars represent the mean ± S.D. of three independent experiments. *P < 0.05. **(G)** Western blot analysis of the effect of overexpression or silencing MAPK8IP1P2 on NF2, RASSF1 and RASSF5 expression in the indicated cells. α-Tubulin served as the loading control. **(H, I)** Real-time PCR **(H)** and Western blot **(I)** analysis of the effect of miR-146b-3p mimics on NF2, RASSF1, and RASSF5 expression in MAPK8IP1P2-overexpressing thyroid cancer cells. Transcript levels were normalized by GAPDH expression. α-Tubulin served as the loading control. Error bars represent the mean ± S.D. of three independent experiments. *P < 0.05. **(J)** The effect of miR-146b-3p mimics on TEAD transcriptional activity was assessed by HOP-Flash luciferase reporter in MAPK8IP1P2-overexpressing thyroid cancer cells. Error bars represent the mean ± S.D. of three independent experiments. *P < 0.05.

Accumulating studies have reported that lncRNAs can serve as competitive endogenous RNAs (ceRNAs) to de-repress miRNA-targeted mRNA expression (56, 57). Therefore, we further explored the potential binding miRNAs of MAPK8IP1P2 by analyzing the correlation of MAPK8IP1P2 with all reported miRNAs in the thyroid cancer dataset from TCGA. As shown in **Figure 6A**, the only miR-146b-3p expression level was negatively correlated with MAPK8IP1P2 expression, and was upregulated in thyroid cancer tissues compared with that in ANT. Using several publicly available algorithms, including miRanda and targetscan, we found that miR-146b-3p had the potential recognition sequences on MAPK8IP1P2, and NF2, RASSF1, and RASSF5 were the potential targets of miR-146b-3p (**Figure 6B**). NF2, RASSF1, and RASSF5 have been reported to promote activity of Hippo signaling by varying mechanism (58–60). Luciferase assay demonstrated that miR-146b-3p mimics suppressed the 3’UTR reporter activity of MAPK8IP1P2, NF2, RASSF1, and RASSF5, but not of the mutant 3’UTR of MAPK8IP1P2 (**Figures 6C, D**). RT-PCR and Western blot analysis revealed that upregulating MAPK8IP1P2 increased, while silencing MAPK8IP1P2 decreased the mRNA and protein levels of NF2, RASSF1, and RASSF5 (**Figures 6E–G**). Importantly, miR-146b-3p mimics not only reversed the NF2, RASSF1, and RASSF5 level enhanced by MAPK8IP1P2 overexpression (**Figures 6H, I**), but also inactivated Hippo signaling in MAPK8IP1P2-overexpressing thyroid cancer cells as indicated by elevated the luciferase reporter activity of HOP-Flash (**Figure 6J**). Thus, these results indicate that MAPK8IP1P2 activates Hippo signaling by sponging miR-146b-3p to disrupt the inhibitory effect of miR-146b-3p on NF2, RASSF1, and RASSF5 expression in thyroid cancer.

### Upregulating MAPK8IP1P2 Inhibits Anoikis Resistance by Sponging miR-146b-3p

**Figure 7:**
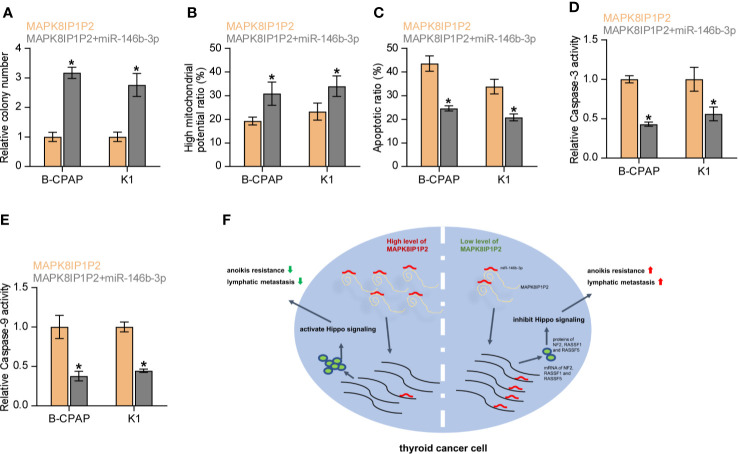
Upregulating MAPK8IP1P2 inhibits anoikis resistance by sponging miR-146b-3p. **(A)** The effect of miR-146b-3p mimics on colony-formation ability in MAPK8IP1P2-overexpressing thyroid cancer cells. Error bars represent the mean ± S.D. of three independent experiments. *P < 0.05. **(B)** The effect of miR-146b-3p mimics on mitochondrial potential in MAPK8IP1P2-overexpressing thyroid cancer cells. Error bars represent the mean ± S.D. of three independent experiments. *P < 0.05. **(C)** The effect of miR-146b-3p mimics on apoptotic ratio in MAPK8IP1P2-overexpressing thyroid cancer cells. Error bars represent the mean ± S.D. of three independent experiments. *P < 0.05. **(D, E)** The effect of miR-146b-3p mimics on caspase-3 **(D)** and caspase-9 **(E)** in MAPK8IP1P2-overexpressing thyroid cancer cells. Error bars represent the mean ± S.D. of three independent experiments. *P < 0.05. **(F)** Hypothetical model illustrates the role and underlying mechanism of MAPK8IP1P2 in lymphatic metastasis of thyroid cancer by miR-146b-3p/Hippo signaling axis.

We further investigated whether miR-146b-3p mediates the effect of MAPK8IP1P2 on anoikis resistance in thyroid cancer cells. As shown in **Figures 7A, B**, miR-146b-3p mimics enhanced the anchorage-independent growth capability and mitochondrial potential in MAPK8IP1P2-overexpressing thyroid cancer cells. In contrast, our results further revealed that miR-146b-3p mimics attenuated the stimulatory effects of MAPK8IP1P2 overexpression on the apoptotic ratio and activity of caspase-3 or -9 in thyroid cancer cells (**Figures 7C–E**). Collectively, our results demonstrate that MAPK8IP1P2 inhibits anoikis resistance by sponging miR-146b-3p in thyroid cancer cells (**Figure 7F**).

## Discussion

The critical findings of the current study present novel insights into the pivotal role of MAPK8IP1P2 in lymphatic metastasis of thyroid cancer by the miR-146b-3p/Hippo signaling axis. Here, we reported that MAPK8IP1P2 was dramatically downregulated in thyroid cancer tissues, especially in those with lymph node metastasis. Gain and loss of function assays demonstrated that upregulating MAPK8IP1P2 inhibited, while silencing MAPK8IP1P2 promoted anoikis resistance *in vitro* and lymphatic metastasis of thyroid cancer cells *in vivo*. Our results further revealed that upregulating MAPK8IP1P2 activated Hippo signaling by disrupting the repressive effect of miR-146b-3p on NF2, RASSF1, and RASSF5 expression by sponging miR-146b-3p as ceRNA, which further suppressed anoikis resistance in thryoid cancer cells. Therefore, our results unravel a novel mechanism by which MAPK8IP1P2 inhibits the anoikis resistance and lymphatic metastasis of thyroid cancer cells, determining the tumor-suppressive role of MAPK8IP1P2 in lymphatic metastasis of thyroid cancer.

As a kind of versatile non-coding RNA, lncRNAs have been extensively validated to function their biological role *via* varying mechanisms (21, 22), in which functioning as ceRNA to sponge target miRNAs to disrupt the miRNAs-mediated degradation of target genes seizes great momentum (56, 57) to be implicated in tumor progression and metastasis (27, 61). Importantly, miRNA-mediated lncRNA/mRNA crosstalk plays an important role in the development and metastasis of thyroid cancer (28, 62, 63), including lymphatic metastasis (32–34). In this study, our results revealed that MAPK8IP1P2 functioned as ceRNA to sponge miR-146b-3p, which further disrupted the inhibitory effect of miR-146b-3p on NF2, RASSF1, and RASSF5 expression. MAPK8IP1P2-mediated this lncRNA/mRNA crosstalk activated Hippo signaling, which further inhibited anoikis resistance and lymphatic metastasis in thyroid cancer. Therefore, our findings uncover a novel mechanism by which MAPK8IP1P2 inhibits the anoikis resistance and lymphatic metastasis of thyroid cancer cells.

Loss or downregulation of core components of Hippo signaling contributes to inactivation of Hippo signaling, which contributes to tumor progression and metastasis. For example, deficiency or inactivation of NF2, which functions to initiate and orchestrate the Hippo pathway (58), has been reported to be a frequent tumorigenic event in several cancer types (64–66). Furthermore, the upstream regulator of the Hippo pathway, ras association domain family (RASSF), suppresses cancer tumorigenesis (67, 68) by regulating MST1/2 activity (59, 60). However, how these regulators of the Hippo signaling are simultaneously disrupted in cancers, leading to constitutively inactivation of Hippo signaling, remains unclear. In this study, our results revealed that the relieved function of MAPK8IP1P2 as a ceRNA to sponge miR-146b-3p upregulated miR-146b-3p. Overexpression of miR-146b-3p directly targeted NF2, RASSF1, and RASSF5 in thyroid cancer cells and inactivated Hippo signaling, which further promoted anoikis resistance and lymphatic metastasis of thyroid cancer. Collectively, our findings clarify that MAPK8IP1P2 activates Hippo signaling by sponging miR-146b-3p to disrupt the inhibitory effect of miR-146b-3p on NF2, RASSF1, and RASSF5 expression in thyroid cancer.

Several lines of evidence have reported the pivotal role of anoikis resistance in lymphatic metastasis of cancer (10, 11), even in lymphatic metastasis of thyroid cancer (18–20). Furthermore, anoikis resistance was reported to be significantly correlated with positive lymph node metastasis in various cancers (12–17). In this scenario, multiple signaling pathways have been demonstrate to promote anoikis resistance, including TGF-β, PI3K/AKT, and Hippo signaling, where the role of Hippo signaling in inducing anoikis resistance gains more attention (9, 50, 51). Importantly, inactivation of Hippo signaling has also been reported to promote lymphatic metastasis of cancers (52, 53). However, the effect of Hippo signaling on lymphatic metastasis of thyroid cancer remains unclear. In the current study, our results showed that MAPK8IP1P2 activated Hippo signaling by sponging miR-146b-3p to disrupt targeting effect of miR-146b-3p on NF2, RASSF1, and RASSF5, which inhibited anoikis resistance and lymphatic metastasis of thyroid cancer. Collectively, our findings provide experimental evidence to support the critical role of Hippo signaling lymphatic metastasis of thyroid cancer.

In summary, our results demonstrate that MAPK8IP1P2 activates Hippo signaling by sponging miR-146b-3p as a ceRNA to disrupt the inhibitory effect of miR-146b-3p on NF2, RASSF1, and RASSF5 expression, which further suppresses lymphatic metastasis of thyroid cancer. Therefore, our results provide novel insights into the underlying mechanism by which MAPK8IP1P2 inhibits lymphatic metastasis in thyroid cancer, supporting the notion that MAPK8IP1P2 can be used as a lymph node metastatic marker in thyroid cancer.

## Data Availability Statement

The original contributions presented in the study are included in the article/supplementary materials. Further inquiries can be directed to the corresponding author.

## Ethics Statement

The studies involving human participants were reviewed and approved by The Institutional Research Human Ethics Committee. The patients/participants provided their written informed consent to participate in this study. The animal study was reviewed and approved by The Institutional Research Animal Ethics Committee.

## Author Contributions

XL and HS conceived the project and drafted the manuscript. QF, JZ, and XL conducted the experiments and contributed to the analysis of data. CL, SL, and YZ performed the animal experiments. NL, LF, and YZ analyzed the informatics data. SL, JX, YF, and XL performed IHC and the analysis of data. XB and JZ conducted the patient information’s organizing. SL and NL contributed to the cell biology and molecular biology experiments. XL and GD edited and revised the manuscript. All authors contributed to the article and approved the submitted version.

## Funding

This study was granted by the National Natural Science Foundation of China (grant number 81702652, 81972499) and the Department of Science and Technology of Jilin Province, China (grant number 20200201181JC).

## Conflict of Interest

The authors declare that the research was conducted in the absence of any commercial or financial relationships that could be construed as a potential conflict of interest.
